# A New 3-Dimensional Dynamic Quantitative Analysis System of Facial Motion: An Establishment and Reliability Test

**DOI:** 10.1371/journal.pone.0113115

**Published:** 2014-11-12

**Authors:** Guodong Feng, Yang Zhao, Xu Tian, Zhiqiang Gao

**Affiliations:** Department of Otolaryngology, Peking Union Medical College Hospital, Chinese Academy of Medical Sciences and Peking Union Medical College, Beijing, China; Xiamen University, China

## Abstract

This study aimed to establish a 3-dimensional dynamic quantitative facial motion analysis system, and then determine its accuracy and test-retest reliability. The system could automatically reconstruct the motion of the observational points. Standardized T-shaped rod and L-shaped rods were used to evaluate the static and dynamic accuracy of the system. Nineteen healthy volunteers were recruited to test the reliability of the system. The average static distance error measurement was 0.19 mm, and the average angular error was 0.29°. The measuring results decreased with the increase of distance between the cameras and objects, 80 cm of which was considered to be optimal. It took only 58 seconds to perform the full facial measurement process. The average intra-class correlation coefficient for distance measurement and angular measurement was 0.973 and 0.794 respectively. The results demonstrated that we successfully established a practical 3-dimensional dynamic quantitative analysis system that is accurate and reliable enough to meet both clinical and research needs.

## Introduction

Facial motion is basic to emotion expression, and impairment to the facial nerves deeply influences physical and emotional well-being. The annual incidence of Bell's palsy is reported to be between 11 and 53.3 cases per 100,000 people [1], [2]. However, the lack of an accurate and objective grading system hinders the development of diagnosis of and research into facial palsy. Clinical research is quantitative and requires fixed written protocols [3].

Facial grading systems can be generally divided into subjective and objective systems [4]. The most widely known and applied facial grading system, the House-Brackmann Grading System, is a typical subjective grading system. It was recommended as the gold standard by the Facial Nerve Committee of the American Academy of Otolaryngology–Head and Neck Surgery [5]. Subjective grading systems are convenient and rapid, but their inherent roughness, intra-rater and inter-observer biases make them inappropriate for comparing the effects of treatment [6]. Objective grading systems can be classified into 2-dimensional and 3-dimensional analysis systems. Two-dimensional analysis systems are much more reliable and objective than subjective grading systems however, they only reflect the projection of motion on a plane [7]. Gross et al [8] compared three-dimensional and two-dimensional analysis systems in analyzing deviation distance. The results demonstrated that 3-dimensional analysis systems were much more accurate than 2-dimensional systems, especially in estimating the lower facial area (2-dimensional system underestimated by 43%).

In recent years, with the development of 3-dimensional object retrieval and transmission techniques [9], [10], we are increasingly able to obtain facial motion data with more accuracy. It is even possible to establish a connection between one's facial expression and affection by fusing the spatial-temporal features [11]. The purpose of this study was to establish a 3-dimensional dynamic quantitative facial motion analysis system and to assess its reliability and accuracy.

## Materials and Methods

Ethical approval was obtained from the Peking Union Medical College Hospital Ethics Committee (No.S059) prior to the study. Written informed consent was obtained from all participants prior to measurement

**Figure 1:**
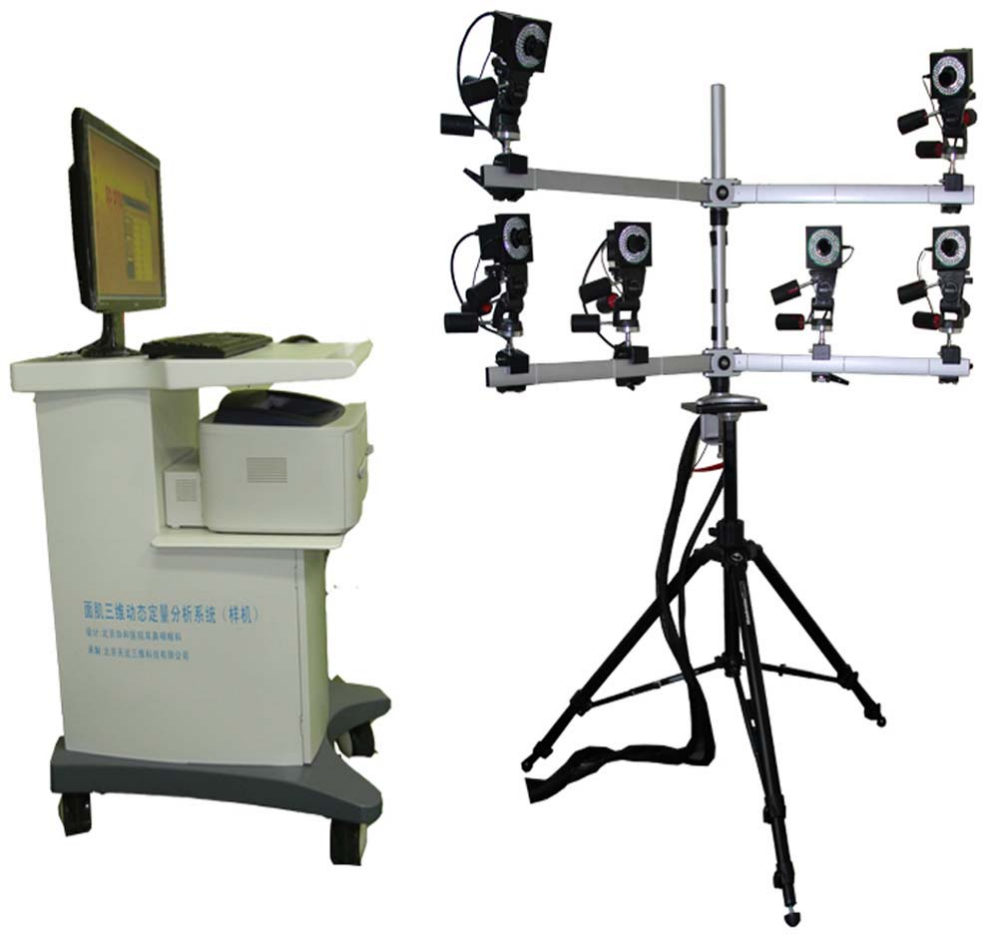
3-Dimensional Dynamic Quantitative Analysis System of Facial Motion.

The system (Fig. 1) consisted of:

- 6 sets of Evision CCD 1360 cameras, with capture frequency of 100 frames per second.
- A multi-channel synchronous controller to synchronize the captures from the cameras.
- A giganet unit connecting all the cameras to the control computer.
- Reflective points with diameters of 3 mm.
- An integrated software package (3DMoCap).
- A T-shaped calibration rod and an L-shaped calibration rod, with four retro-reflective markers at a given distance.
- A helmet made to order.

Six cameras were placed in the shape of a symmetrical “L” on a tripod to ensure that every reflective point on the participant could be detected by at least three cameras. Infrared light, emitted from the circular LED lights around the lenses, was captured by the cameras. The light information was converted to electrical information and then transmitted to the data processing module. Before measurement, the system had to be calibrated to ensure precise connections between each camera. A T-shaped rod with 3 reflective points in a straight line was moved horizontally and vertically in the field of view of the 6 cameras. The spatial location and spatial relationship of the 3 reflective points were known and were input into the system before calibration. Based on data from a preliminary experiment, at least 3000 frames were required for each camera. After calibration, the 3DMoCap software automatically calculated the calibration systemic error, which had to be less than 0.3 mm. Errors of more than 0.3 mm often resulted from rapid movements of the rod. During measurement, the 3-dimensional signals captured by the cameras were matched to the reference database established during calibration. Then, the spatial location of each landmark in every frame and the displacement of each landmark between every frame could be estimated. Since the capture frequency was fixed, the velocity and acceleration could be calculated.

### Measurement of accuracy

To measure the accuracy of the system, a linear rod 176.84 mm in length and a rectangular rod with a 90 degree angle were used. To ensure the stability of every test, we used prime lenses whose focal length were fixed. Although the theoretical focal length of every lens was known, the optimum working distance of the six lenses was not obtainable before the test. First, the linear rod with two reflective points at the given distance (176.84 mm) was moved from 60 cm to 90 cm away from the center of the cameras. The system would display the length measured. The distance which showed the closest result was thought to be the optimum measurement distance. Having found the optimum measurement distance, the rods were used to measure the static and dynamic accuracy of the system. To measure the static accuracy, two rods were placed on a table in front of the cameras for around 5 seconds. To obtain the dynamic accuracy, two rods were moved horizontally and vertically in front of the cameras for around 1 minute. For each test, we randomly picked at least 10 frames. In each frame, the end-to-end distance of the linear rod and the angle of the rectangular rod were calculated automatically. Then, the average error was calculated.

Nineteen healthy volunteers were recruited to evaluate the reliability of the system. Inclusion criteria for the study stipulated an age range of 18 to 30 years, no history of facial paralysis or other relevant medical history.

### Observational points

**Figure 2:**
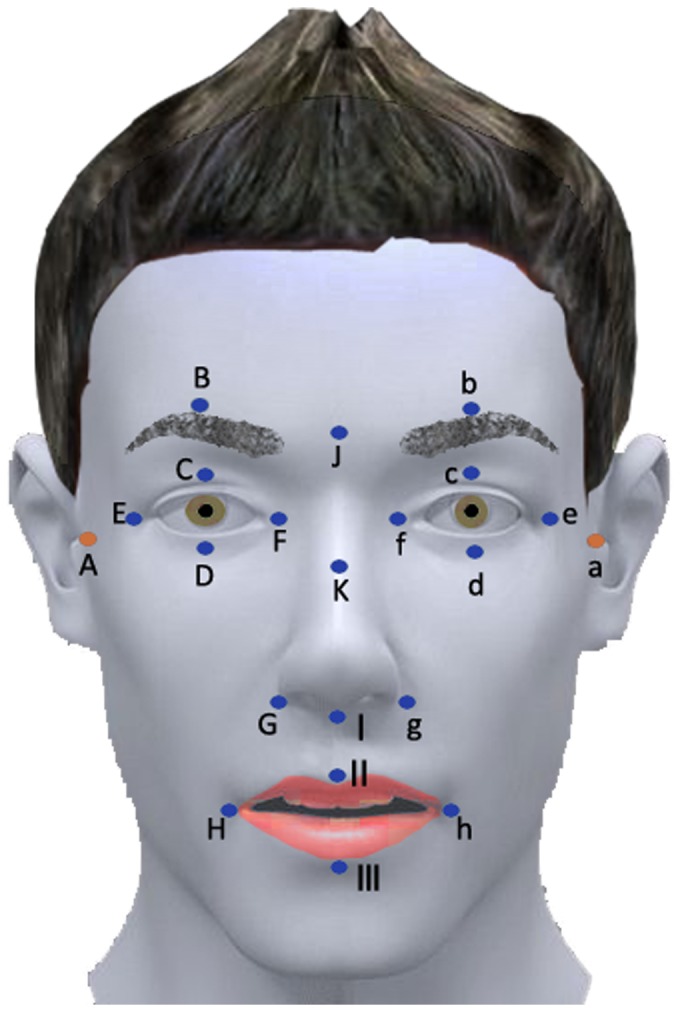
Observational points on face. A/a tragus parallel to the upper wall of external acoustic canal, B/b central position above the eyebrow, C/c center of the upper eyelid, D/d center of the lower eyelid, E/e angulus oculi temporalis, F/f angulus oculi medialis, G/g ala of the nose, H/h corner of the mouth, I root of the columella nasi, J center of eyebrows, K bony–cartilaginous junction along the nasal dorsum, II philtrum, III center of the lower lip.

The observational points should be both concise and comprehensive, and should take the anatomical landmarks on face into consideration. Referring to the House-Brackmann grading system and the method of Hontanilla and Tzou, we chose 21 observational points on the face (Fig. 2) (A/a tragus parallel to the upper wall of external acoustic canal, B/b central position above the eyebrow, C/c center of the upper eyelid, D/d center of the lower eyelid, E/e angulus oculi temporalis, F/f angulus oculi medialis, G/g ala of the nose, H/h corner of the mouth, I root of the columella nasi, J center of eyebrows, K bony-cartilaginous junction along the nasal dorsum, II philtrum, III center of the lower lip).

The disposable reflective markers we used were 3 mm in diameter, the weight of which could be ignored.

### Reference coordinate system

**Figure 3:**
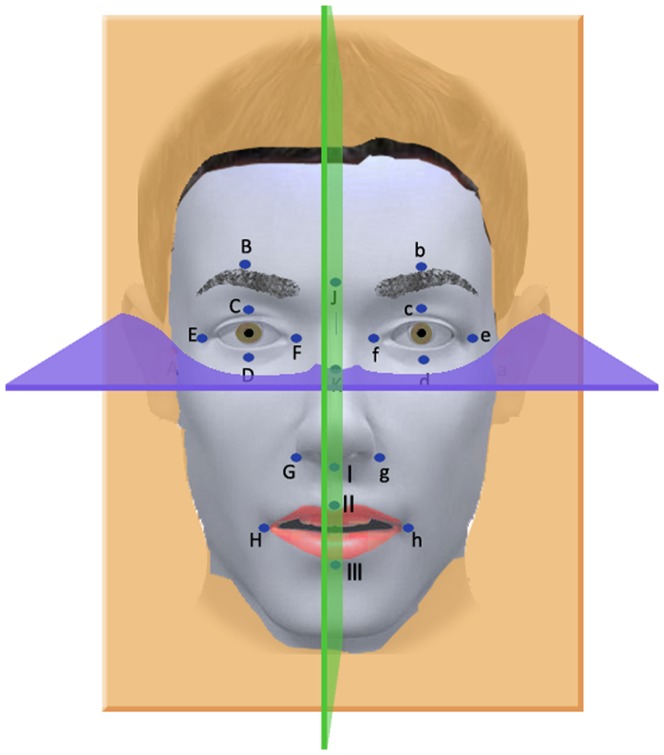
Reference planes.

To describe the direction of the points accurately, we defined three reference planes (Fig. 3). The horizontal plane ran through the tragus points and the nasal center point. The sagittal plane was established perpendicular to the horizontal plane and through the center of the nose. The coronal plane was established through the left tragus point and perpendicular to both the horizontal and sagittal planes. The 3DMoCap software automatically identified the markers on the face and established the reference planes above. We also defined moving directions as follows: moving towards the cameras is “forward”, and moving away from the cameras is “backward”; with reference to the horizontal plane (Y axis), moving upward is “+” and moving downward is “–”; with reference to the sagittal plane (X axis), moving left is “+” and moving right is “–”.

### Reference helmet

**Figure 4:**
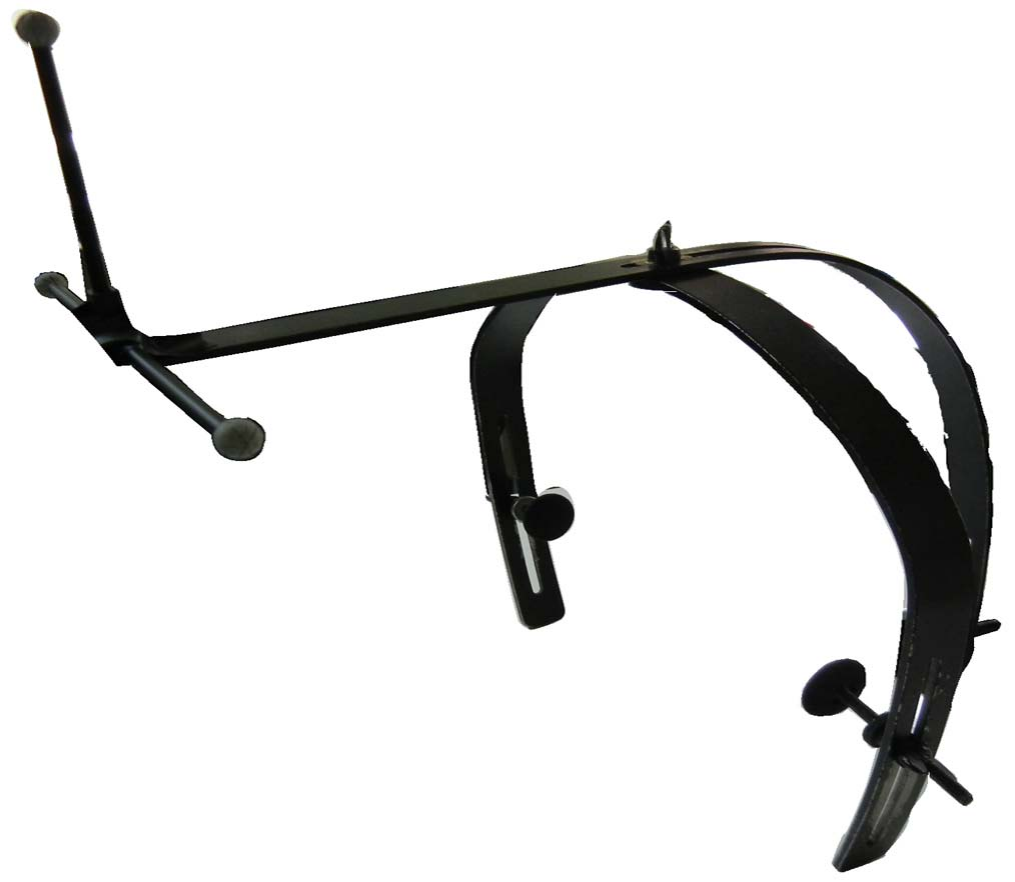
Specialized helmet.

We designed a helmet to cater to clinical requirements (Fig. 4). The helmet was made of titanium alloy and weighed nearly 300 g. There were three reflective points positioned at the front end of the sagittal branch, which worked as an “absolute” reference coordinate system. The dynamic parameters were calculated in reference to the coordinate system. The lateral end of the helmet was firmly attached to the skin above the temporal bone behind the ear. The posterior end of the helmet was firmly attached to the occipital protuberance.

### Measuring procedure

**Figure 5:**
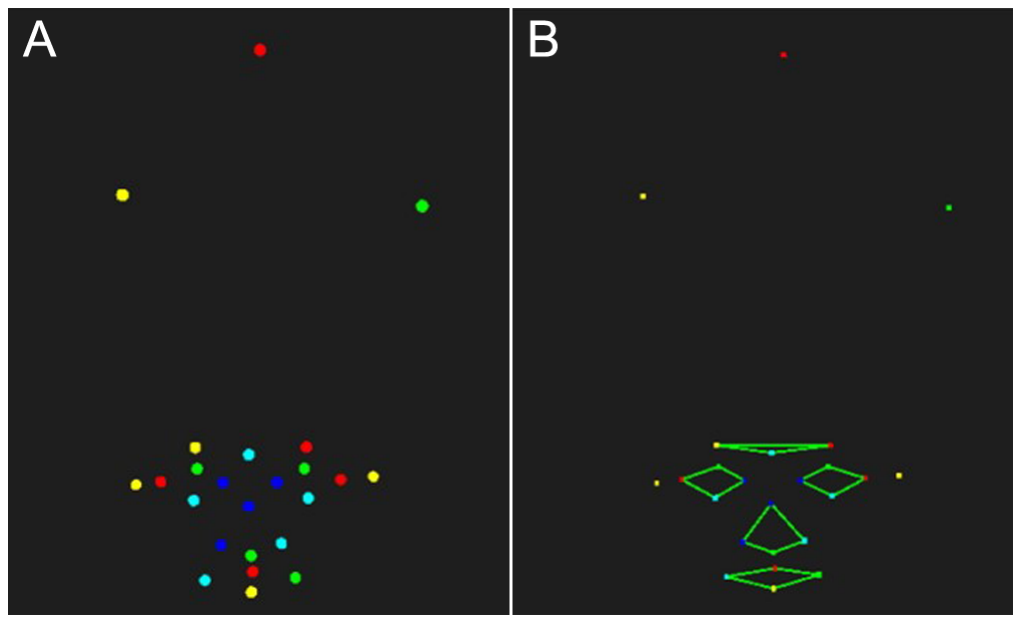
A, online reconstruction; B, offline reconstruction.

To minimize error, participants were asked to remove any reflective objects on their head or neck before the test. Adhesive reflective points were placed precisely in positions on the resting face. Then, participants were seated comfortably and vertically on a chair in front of the cameras, which were continuously monitored by the system. Before the test, the helmet was firmly fixed to the head. The helmet moved simultaneously with the head, making it unnecessary to keep the head still during the test procedure. After all the preparations were done, participants were instructed to perform 10 standardized facial movements (Table 1) following verbal instructions from an experimenter. The software simultaneously reconstructed the markers' trajectory in the virtual coordinate system (Fig. 5A). Subsequently, the software 3DMoCap calculated the parameters of each facial movement in a set pattern. It took only 58 seconds to perform the whole measurement. The overall time from sticking the reflective points to outputting the result was less than 5 minutes for a single participant. When the first test was completed, all the markers were removed from the participant's face. After 10 minutes, the above procedure, from sticking to removing markers, was repeated to determine the test-retest reliability of the system.

**Table 1:** Standardized facial expressions.

Standardized facial expressions
1.Maximal brow lift
2.Light eye closure
3.Maximally tight eye closure
4.Maximally frown
5.Maximally showing teeth
6.move right mouth corner to right-back
7. move left mouth corner to left-back
8. maximal jaw drop
9.Smile(showing at least 8 upper teeth)
10.Maximal pucker

## Results

### Accuracy

Measuring the same object from different places gave different results. A rod 176.84 mm in length had results ranging from 175.64 mm to 178.36 mm while the measuring distance between the cameras and the rod extended from 60 to 90 cm. We also found that the measuring results decreased with the increase of distance between the cameras and objects, 80 cm of which was considered to be optimal.

The average distance measuring error was 0.19 mm and the average angular measuring error was 0.29°. The mean dynamic measuring result for the linear rod was 175.75±0.196 mm, which was not significantly different from the reference value (*t* = 1.51, *p* = 0.165>0.05). This was also the case for the rectangular rod, the mean result of which was 88.96°.

### Reliability

The testers were not exposed to any invasive harm. The intra-class correlation coefficient of static movement is shown in Table 2. The average intra-class correlation coefficients (*ICC*) for the static distance and angle measurements were 0.973 and 0.794 respectively. In measuring dynamic movement, the maximum *ICC* was 0.999, while the minimum was 0.11. It was interesting to find that the reliability of the upper face movement was commonly higher than for the lower face. Generally speaking, the measurement parameters concerning maximum deviation distance, maximum speed and maximum acceleration rate were very reliable.

**Table 2:** Static measurement *ICC*.

static measurement	*ICC*	95%*CI*	
G-H (distance)	0.995	0.986	0.998
G-H (direction) X	0.643	0.288	0.844
G-H (direction) Z	0.346	−0.106	0.682
g-h (distance)	0.985	0.962	0.994
g-h (direction) X	0.814	0.586	0.924
g-h (direction) Z	0.706	0.39	0.875
I-G (distance)	0.986	0.965	0.995
I-G (direction) X	0.585	0.2	0.815
I-G (direction) Z	0.721	0.416	0.882
I-g (distance)	0.988	0.97	0.995
I-g (direction) X	0.11	−0.343	0.525
I-g (direction) Z	0.727	0.427	0.885
∠CED	0.985	0.962	0.994
∠ced	0.819	0.596	0.926
∠EHh	0.745	0.458	0.893
∠ehH	0.627	0.263	0.837
H-h(distance)	0.982	0.956	0.993
E-H(distance)	0.986	0.964	0.994
E-h(distance)	0.987	0.967	0.995
II-III (distance)	0.951	0.874	0.98

X =  X axis, Z =  Z axis, ∠ = angle.

## Discussion

As more and more research focuses on facial paralysis treatment, there is an increasing need for a reliable and objective grading system. Theoretically, a 3-dimensional dynamic quantitative analysis system is able to provide static and dynamic parameters such as moving direction, velocity, and acceleration. Analyzing these parameters makes it possible to accurately assess the symmetry of the face, and thereby assess the function of the facial nerve. In recent years, dynamic motion capture techniques have been widely used in areas like animation, video games, and ergonomics. Due to their intrinsic non-contact properties, they have been used by several researchers to assess facial nerve function [3], [12]–[15]. However, several issues have not yet been solved.

1. The necessity of using marker points. Some researchers tried to record the changes of both geometric shapes and the positions of objects in real time, and then synthesize three-dimensional geometry videos [16]. However, there was no absolute correspondence between different frames, and a large amount of data needed to be processed. Marker-based facial tracking has been used for many years, the accuracy of which can reach up to 0.01 mm/m [17]. Because of the limitations of computer-based automated identification, realizing accurate three-dimensional measurement still relies on marker balls or a disk. We made some changes to the mounting method to improve the accuracy of the system. The adhesive marker discs we used were 3 mm in diameter, light and flat, and it took only one minute to attach all the points. In the process of facial motion, all the reflective points could be detected by the system and the facial expression was not influenced by the marker discs.
2. How to determine a reference point. To analyze a three-dimensional movement, at least three static points are necessary; the tragus points and the lower jaw are common choices [18]. Several studies have revealed that no points on the face are static [19], [20]. One solution was to fix a device to the maxillary teeth to work as a reference system [21], another way was to separate the face into two parts and each part worked as the other part's reference system [14]. Unfortunately, the reference system they adopted tended to deviate during the motion of facial expressions. It seems impossible to find one static point on the face. In our study, we designed a specialized helmet on the basis of anatomical information on Chinese people. The anterior branch of the helmet has 3 reflective markers attached. Through the 3 markers, an origin and a plane could be determined. Then, a line that was both perpendicular to the plane and crossing the origin could help establish a 3-dimensional coordinate system. Thus, we could establish a stable 3-dimensional coordinate system using only 3 points. The three supporting points extended from the helmet were fixed to a participant's head firmly, even during the motion of shaking their head. To minimize the influence of facial expression, there should be as few skin contact points as possible. Thus, we only chose three contact points, which was the least possible but enough to support the helmet over the head. Before the test, we were concerned that the extra helmet steadiness might be at the cost of comfort. However, the feedback from the participants was not negative.
3. The design for measuring parameters is controversial. The stable geometrical relationship of different facial parts is the basis of face identification, which is hard to describe. Tzou [12] demonstrated a series of trajectories, but no detailed measuring parameters and their meanings were provided. The angles provided by Hontanilla [14] were merely the angles between different observational points, which did not take the direction of muscular movement into consideration. We provided a descriptive approach that was on the basis of stereoscopic anatomy. We used 3 anatomical planes to describe direction. In our previous study, we found that the three planes could accurately establish a 3-dimensional image of skull base structures [22]. We chose 21 observational points, as mentioned in the method section. Each point was measured for moving direction, distance, velocity, acceleration, maximal velocity and maximal acceleration, which were necessary for a comprehensive analysis of facial motion.
4. There is a gap between facial movement and facial nerve function. Both the severity of the facial nerve lesion and the amount of facial nerve excitation can have an influence on facial muscle movements. Thus, it is theoretically possible to judge the function of the facial nerves using a series of measurement indexes. Frey [20] raised a conception that could tell the status of the facial nerves by measuring the deviation of the observational points during facial motion. However, the lack of further study and the inappropriate design of measurement indexes limited the application of this method. Salgado's 3D methods allowed for accurate study of facial volume and facial movement and the forces generated within tissues [13], but there was no applicable method available in their study. It still remains debatable which points best reflect the motion of different facial regions. According to Newton's second law of motion, coupling force and acceleration, F  =  ma, once the mass of a certain point is fixed, the acceleration is positively correlated with force. In our study, the acceleration could be easily obtained, making it possible to assess the function of the facial nerves and facial muscles.

To meet the requirements of clinical assessments and research, the measurement of facial nerve function should be consistent, reliable and quantitative. With the advent of advanced imaging technology, the possibility of accurately measuring facial motion is becoming more and more promising.

## Conclusion

We established a practical 3-dimensional dynamic quantitative analysis system. Generally speaking, it was accurate and reliable enough to meet clinical and research needs. Several parameters concerning directions of facial landmarks were not satisfactory. One reason might be inconsistency between repetitive facial motions. Further study will be focused on analyzing patients' facial movements.
